# Dual-Crosslinked Alginate-Based Hydrogels with Tunable Mechanical Properties for Cultured Meat

**DOI:** 10.3390/foods11182829

**Published:** 2022-09-13

**Authors:** Irfan Tahir, Rachael Floreani

**Affiliations:** 1 Department of Mechanical Engineering, University of Vermont, Burlington, VT 05405, USA; 2 Department of Mechanical Engineering, Department of Electrical and Biomedical Engineering, Materials Science and Engineering Graduate Program, Food Systems Graduate Program, University of Vermont, Burlington, VT 05405, USA

**Keywords:** alginate, cultured meat, muscle cells, scaffolds, mechanical tunability, tissue engineering

## Abstract

Cultured meat refers to the production of animal tissue by utilizing the same techniques as tissue engineering through cell culture. Various biomaterials have been designed to serve as in vitro supports for cell viability, growth, and migration. In this study, visible light and dual-crosslinked alginate hydrogels were designed to enable control of the physical and mechanical properties needed for the fabrication of cultured meat scaffolds. We hypothesized that a difference in hydrogel stiffness would influence cell behavior, indicating the efficacy of our processing methods to benefit the cultured meat field. Herein, we synthesized and created: (1) methacrylated alginate (AlgMA) to enable covalent crosslinking via visible light exposure, (2) Methacrylated alginate and arginyl-glycyl-aspartic acid RGD conjugates (AlgMA-RGD), using carbodiimide chemistries to provide cell-binding sites on the material, and (3) designer hydrogels incorporating different crosslinking techniques. The material and mechanical properties were evaluated to determine the structural integrity of the hydrogels, and in vitro cell assays were conducted to verify cytocompatibility and cell adhesion. Gelation, swell ratio, and weight loss calculations revealed longer gelation times for the AlgMA scaffolds and similar physical properties for all hydrogel groups. We showed that by adjusting the polymer concentration and the crosslinking methodology, the scaffold’s mechanical properties can be controlled and optimized within physiological ranges. Incorporating dual crosslinking significantly increased the compressive moduli of the AlgMA hydrogels, compared to visible-light crosslinking alone. Moreover, the muscle satellite cells responded favorably to the AlgMA scaffolds, with clear differences in cell density when cultured on materials with significantly different mechanical properties. Our results indicate the usefulness of the dual-crosslinking alginate hydrogel system to support in vitro meat growth.

## 1. Introduction

Cultured meat is the production of animal tissue in vitro, a process that falls under the broader field of tissue engineering. Traditionally, tissue engineering techniques were limited to applications in medicine, where functional tissues are created to help restore, repair or replace damaged human tissue and organs [1]. The same techniques are used to produce cultured meat, yet many technical challenges remain before cultured meat products can become mainstream [2]. One of these challenges is accurately portraying the tissue’s extracellular matrix (ECM); for example, it is imperative that the cell scaffold structure be three-dimensional (3D), and contain an interconnected network embedded in a viscoelastic material. The ECM regulates the cell’s dynamic behavior, provides structural support, and relays information between cells. Various biomaterials have been designed to recapitulate the ECM to serve as in vitro supports for cell viability, growth, and migration. The literature has shown that successful scaffolds need to exhibit the appropriate material and mechanical properties to support engineered tissue [3,4,5,6,7]. One type of biomaterial that has proven successful in 3D cell-culture studies is a hydrogel made from alginate [8,9]. 

Alginate has several key benefits for in vitro meat culture. Alginate is (1) derived from brown algae and is sustainable, (2) readily available, and (3) ideal for making hydrogel scaffolds [8,10,11]. Introducing a divalent cation to sodium alginate enables the formation of a 3D network via ionic crosslinking between carboxyl groups on the alginate backbone. Alginate hydrogels are recognized for their physicomechanical properties, which can be fine-tuned by adjusting the polymer’s molecular weight, chemical composition, and method of crosslinking [12]. Ionic crosslinking is convenient and quick; however, the bonds are reversible and less stable than covalent bonds. To overcome this drawback, alginate can be chemically functionalized to enable covalent crosslinking, forming a more stable network structure [13]. Recently, our group has developed the technology to incorporate visible green crosslinking into a dual-crosslinked system, wherein alginate is chemically modified to undergo both ionic and covalent crosslinking [14,15]. Individual or simultaneous adjustments in the formation of ionic and covalent bonds can be used to control the mechanical properties of the materials, including gelation time, stiffness, and strength [16,17,18,19]. For example, the stiffness of alginate can be fine-tuned to better replicate that of skeletal muscle tissue, which is typically in the range of approximately 16–60 kPa [20,21,22]. Photo-crosslinking via the methacrylate functional group serves as an established model system to fine-tune the hydrogel’s mechanical properties and to conduct robust research. Herein, we incorporate our visible green-light crosslinking method in addition to crosslinking with salts. 

Despite these beneficial characteristics, alginate inherently lacks cell adhesion motifs [10]. To fill this gap, cell-adhesive biomolecules can either be conjugated covalently onto the alginate base ingredient or blended with alginate, resulting in bioactive hydrogels that are ideal for tissue engineering [23]. Arginyl-glycyl-aspartic acid (RGD) conjugation, a ligand that promotes cell adhesion, significantly increases cell proliferation and migration on alginate scaffolds and is often used as a model system [24,25]. Thus, by using a model system based on our own work and using established methods from the literature, we created a material that supported cell adhesion to monitor cell viability and density via RGD modification and provided the ability to make materials with significantly different mechanical properties via methacrylation and photo-crosslinking.

Fabricating dual-crosslinked alginate hydrogels, which display controlled mechanical properties, is a promising avenue for cultured meat production. We hypothesized that muscle cells would respond differently to materials with significantly different elastic moduli. Herein, we synthesized: (1) methacrylated alginate (AlgMA) to enable covalent crosslinking via visible light exposure, (2) RGD conjugates (AlgMA-RGD) using carbodiimide chemistries to provide cell-binding sites on the material, and (3) designer hydrogels incorporating different crosslinking techniques. We are the first to apply this model system to investigate the potential for muscle tissue engineering in manufacturing cultured meat. The material and mechanical properties were evaluated to determine the structural integrity of the hydrogels, and in vitro cell assays were conducted to verify that the hydrogels are not toxic to myoblasts and determine the effect of material stiffness on cell adhesion and density. These materials were fabricated to evaluate our hypothesis in a short-term, proof-of-concept study, using mechanical properties to control cell response for the design and optimization of cultured meat scaffolds. 

## 2. Materials and Methods

### 2.1. Materials

Sodium alginate (Manugel, MW_v_ ≈ 170–240 kDa) was generously donated by FMC Biopolymer (Philadelphia, PA, USA). C2C12 murine myoblasts (catalog #91031101), phosphate-buffered saline (PBS), calcium chloride (CaCl_2_), methacrylic anhydride (MA), sodium hydroxide (NaOH), hydrochloric acid (HCl), N-ethyl-N′(3-dimethyl aminopropyl) carbodiimide hydrochloric acid (EDC), N-hydroxysuccinimide (NHS), eosin Y (photosensitizer), triethanolamine (TEOA, photo-initiator), 1-vinyl-2-pyrrolidinone (1VP, catalyst), and Triton X-100 were purchased from Sigma-Aldrich (St. Louis, MO, USA). Cysteine-L-arginyl–glycyl-L-aspartic acid (cRGD) was purchased from Genscript (Piscataway, NJ, USA). A WST-8 Cell Proliferation Assay Kit (catalog #KA1385) was purchased from Abnova (Walnut, CA, USA). Deuterium oxide (D_2_O) (catalog #166301000), a Live/Dead Assay Kit (catalog #L3224), Dulbecco’s modified Eagle medium (DMEM) (catalog #11965092), Trypsin-EDTA (catalog #25300062), penicillin-streptomycin (Pen-Strep) (catalog #10378016), and fetal bovine serum (FBS) (catalog #10100147) were purchased from Thermo Fisher Scientific (Waltham, MA, USA). Dialysis tubing ((MWCO = 6–8 kDa, catalog #08700148) was purchased from Spectrum Chemical (New Brunswick, NJ, USA). Nuclear magnetic resonance (NMR) tubes were purchased from DWK Life Sciences (catalog #8971100007, Millville, NJ, USA). 

### 2.2. Chemical Modification of Alginate

#### 2.2.1. Methacrylated Alginate (AlgMA)

Methacrylated alginate (AlgMA) was synthesized as described in the literature [15,25,26]. Briefly, sodium alginate (Alg) was dissolved in deionized (DI) water to create a 1% (*w*/*v*) solution and a 10-fold molar excess of methacrylic anhydride was added to the alginate solution. The pH of the solution was periodically adjusted to 8.5, using 1 N NaOH, and the methacrylation reaction was conducted for 24 h at room temperature. The final pH was adjusted to 7 using 1 N HCl, and the AlgMA solution was purified via dialysis against DI water for three days to remove any nonreacted components. The solution was frozen and lyophilized to obtain a dry polymer and was stored at −20 °C until use.

#### 2.2.2. Alg and AlgMA RGD Conjugation

Alg or AlgMA was dissolved in DI water (1%, *w*/*v*) at room temperature. EDC was added to the AlgMA solution while mixing for 30 min at room temperature, followed by the addition of NHS. The COOH:EDC:NHS molar ratio remained consistent (1:8:3.2) during the carbodiimide reaction, wherein COOH refers to the moles of alginate carboxyl groups [24,25]. cRGD was thawed to room temperature and used as sourced from the supplier; cRGD was added to the functionalized Alg or AlgMA solution. The carbodiimide reaction was conducted for five hours at room temperature. The products, Alg-RGD and AlgMA-RGD, were dialyzed against DI water for three days to remove nonreacted EDC and NHS. The solution was frozen and lyophilized to obtain a dry polymer and stored at −20 °C until use.

#### 2.2.3. Chemical Characterization

A 1% (*w*/*v*) solution of Alg and AlgMA, with and without RGD modification, was prepared in D_2_O to obtain the ^1^H-NMR spectra. The solutions were characterized via proton ^1^H-NMR spectroscopy (Bruker AVANCE III 500 MHz high-field ^1^H-NMR spectrometer (Billerica, MA, USA), collecting 64 scans, at 20 Hz). 

### 2.3. Physical Characterization and Gelation Kinetics 

Rheometry was performed using an AR2000 rheometer (TA Instruments, New Castle, DE, USA) equipped with a Peltier plate to determine the viscosity and gelation kinetics of ionically crosslinked Alg and ionically crosslinked or photo-crosslinked AlgMA (i.e., visible light-activated crosslinking) [14,16]. All tests were performed at 37 °C using a 20-mm diameter 1°59′6″ steel cone geometry with a truncation gap of 57 µm. Polymer solutions were loaded onto the plate and the top geometry was lowered onto the material until an axial load was recorded. Viscosity was measured at shear rates ranging from 1–100 (1/s), over a 120-second time period. Viscosity values were calculated and plotted using TA Data Analysis software (TA Instruments, New Castle, Delaware, USA) (*n* = 4).

The gelation kinetics of the precursor solutions were assessed at 1% oscillatory radial strain and 1 Hz; gelation times were determined as the intercept between the shear storage (G′) and loss moduli (G″). For ionic crosslinking, 1 M CaCl_2_ was sprayed on the test sample after 30 s. To achieve covalent crosslinking between AlgMA or AlgMA-RGD polymer chains, visible green light was used, as described in our previous work [27,28]. Briefly, polymer precursor solutions (1% and 3%, *w*/*v*) were prepared in DI water. Polymer solutions were then mixed with photo-activators to obtain the following concentrations: 1 mM eosin Y, 125 mM TEOA, and 20 mM 1VP. The polymer solutions were exposed to green light after 30 s using a custom light setup (525 nm, custom 9.84-cm diameter light-emitting diode (LED) array, NFLS-G30X3-WHT, Super Bright LEDs) for 15 min. G′ and G″ values were calculated and plotted using TA Data Analysis software.

### 2.4. Scaffold Fabrication and Characterization 

#### 2.4.1. Scaffold Preparation

Ionically crosslinked, photo-crosslinked, and dual-crosslinked hydrogels were formed using 6-mm diameter cylindrical molds (Table 1.). Alg and AlgMA precursor solutions, with and without RGD, were prepared in DI water (1 and 3%, *w*/*v*). Ionically crosslinked hydrogels were prepared by injecting Alg or AlgMA precursor solutions into molds and then stored at −20 °C for two hours. Next, 1 M CaCl_2_ was added dropwise onto the frozen pre-forms. Photo-crosslinked AlgMA-based hydrogels were created by injecting AlgMA precursor solutions, mixed with 1 mM eosin Y, 125 mM TEAO, and 20 mM VP, into molds and exposing them to green light using a custom light setup (vide supra) for 15 min. Dual crosslinking was achieved by combining crosslinking techniques; photo-crosslinking preceded ionic crosslinking. Discs of uniform size were obtained by removing the hydrogels carefully from the molds. For the in vitro cell study, the same process was followed; however, the hydrogels were cast directly in a nontreated 48-well plate. 

#### 2.4.2. Scanning Electron Microscopy (SEM)

Immediately after crosslinking, hydrogel samples were flash-frozen in liquid nitrogen to maintain the internal structure of the scaffold. Once frozen, the samples were lyophilized, cryo-fractured, and sputter-coated with 10 nm of Au–Pd prior to imaging. Scanning electron micrographs (Zeiss Sigma 300 VP Field-Emission SEM, Oberkochen, Germany) were used to qualitatively characterize the internal structure of the crosslinked scaffolds and confirm an interconnected porous network.

#### 2.4.3. Equilibrium Water Content and In Vitro Degradation

The equilibrium water content, i.e., the swell ratio, of each hydrogel group was quantified to analyze the hydrogel material properties. Initial weight measurements of lyophilized samples (W_i_) were recorded (*n* = 4). Next, the samples were immersed in 1 mL PBS and placed in a 37 °C shaker incubator at 150 rpm for 24 h, and the wet weight (W_w_) was recorded. The samples were then lyophilized, and a final dry mass was recorded for each sample (W_d_). Equilibrium water content and weight loss were calculated as follows: equilibrium water content (%) = (W_w_ − W_d_)/W_d_ × 100; weight loss (%) = (W_i_ − W_d_)/W_i_ × 100. 

#### 2.4.4. Unconfined Uniaxial Compression Testing

The unconfined uniaxial compressive moduli of all hydrogel groups, without RGD modification, were determined. Compression tests were conducted using an electromechanical universal testing machine (Test Resources, 200 series, Shakopee, MN, USA) with a 5 kN load cell (Test Resources, model no. SM-5000N-294, Shakopee, MN, USA) and 56-mm diameter compression platens (Test Resources, model no. G23). Cylindrical hydrogel specimens (6-mm diameter × 3-mm thick) (*n* = 3) from each hydrogel group were fabricated and hydrated in DI water for 30 min. Samples were placed on the testing machine and the flat geometry was lowered until a force of 0.01 N was measured and then cleared. The gap height was recorded as the original gauge length for displacement measurements. Hydrogels were subjected to uniaxial compressive loads at 10 mm/min up to 35% compressive strain. Elastic stress and strain values were plotted, and the elastic modulus for each sample was calculated as the slope of a linear fit between 5 and 15% compressive strain, within the linear-elastic region of the stress-strain plots [29].

### 2.5. Cell Culture

A murine myoblast C2C12 cell line was cultured at a density of 5 × 10^3^ cells/cm^2^ in DMEM (high-glucose without sodium pyruvate) cell culture media containing 10% FBS and 1% pen-strep at 37 °C and 5% CO_2_ [29]. To quantify the potential toxicity of our materials to cells, the mitochondrial activity of the C2C12 cells was analyzed using a WST-8 Cell Proliferation Assay. Briefly, hydrogels (*n* = 4) were placed in wells and covered with cell culture media. After 24 h of culture at 37 °C, 5% CO_2_, the leachates from each hydrogel were transferred to a cell-seeded 96 well-plate and cultured for an additional 24 h; non-modified cells served as the positive control. The optical density absorbance was measured at 460 nm using a microplate reader (Biotek Synergy H1, Winooski, Vermont, USA). Absorbance values for cells cultured in leachate solution were normalized to cells in a culture medium without hydrogel leachates. 

To qualitatively characterize cell adhesion and adhesion density on the materials, 40,000 cells were seeded on cylindrical hydrogels (9 mm in diameter × 3 mm thick), comprising Alg and AlgMA, with and without RGD modification, in non-tissue-treated 48-well plates. Cell-seeded hydrogels (*n* = 4) were incubated for 24 h at 37 °C and 5% CO_2_, after which the hydrogels were washed three times with PBS to remove non-adhered cells. Calcein-AM fluorescence dye was added to the wells and incubated at room temperature for 15 min. The hydrogels were imaged with the fluorescence channels for live cells (green, wavelength 525 nm) using a Biotek Cytation 5 (Winooski, Vermont, USA) microscope. 

### 2.6. Statistical Analysis

The mean and standard deviation for each sample group in the physicomechanical analysis and quantitative cell assays were calculated. A one-way analysis of variance (ANOVA) was performed to determine the statistical significance between the sample groups. Additional statistical testing in the form of *t*-tests (two-tailed distribution, assuming unequal variance) was performed to determine the statistical significance (*p* < 0.05) between the sample groups.

## 3. Results and Discussion

### 3.1. Modification of Alginate and Chemical Characterization

**Figure 1:**
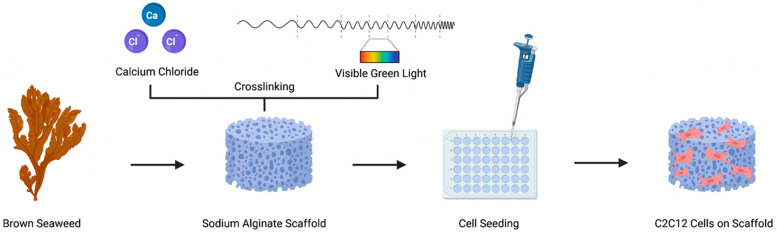
Schematic representation of the alginate-based hydrogel components and processing of scaffolds using ionic, visible light, and dual-crosslinking techniques. The scaffolds were seeded with murine myoblasts (C2C12) to examine the cellular response to various hydrogel samples exhibiting a range of stiffness values.

**Figure 2:**
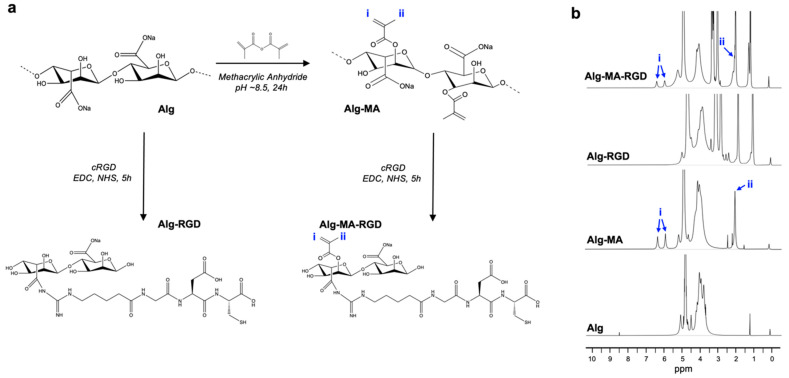
(**a**) Sodium alginate (Alg) was chemically modified with methacrylic anhydride to produce methacrylated alginate (AlgMA). Both Alg and AlgMA were further modified, via carbodiimide chemistry, with the cell adhesion ligand RGD. (**b**) ^1^H-NMR spectra of non-modified and modified alginate with and without RGD conjugation. The notations i and ii indicate the proton peaks of vinyl methylene and methyl groups on the functionalized backbone, respectively.

Alginate was chemically modified and formed into hydrogel scaffolds using ionic and/or visible-light crosslinking. The crosslinked materials were prepared to perform a proof-of-concept study with murine myoblasts (Figure 1), to determine the effect of varying the scaffold’s mechanical properties. While alginate can crosslink to itself using divalent cations, we chemically modified alginate with an acrylate group (Figure 2a) to enable additional covalent crosslinking via visible green light exposure, in the presence of photo-initiators. As alginate by itself does not possess any cell adhesion motifs, Alg and AlgMA were modified to incorporate a cell adhesion ligand, e.g., RGD, using carbodiimide chemistry (Figure 2a) [10,25]. Using ^1^H-NMR analysis, the functionalization of alginate was verified. As shown in Figure 2b, new peaks appeared at 1.9, 5.7, and 6.1 ppm (denoted as i and ii), each of which indicates the proton peaks of vinyl methylene and methyl in the methacrylated group of AlgMA and AlgMA-RGD. The addition of RGD was also verified on both the Alg and AlgMA materials by the presence of peaks between 2.8 and 3.1 ppm. The broad peak at 3.4–4.1 ppm identifies the protons on the non-modified alginate backbone. Indeed, conjugating cell adhesion motifs to the polymer backbone is not always required, especially when the scaffold is made from animal-derived materials, such as collagen and gelatin. In some cases, nonmodified alginate scaffolds have supported cell adhesion in short-term culture conditions [30,31,32]; however, cell adhesion on nonmodified alginate was significantly lower compared to gelatin [32].

### 3.2. Physical Characterization and Gelation Kinetics

**Figure 3:**
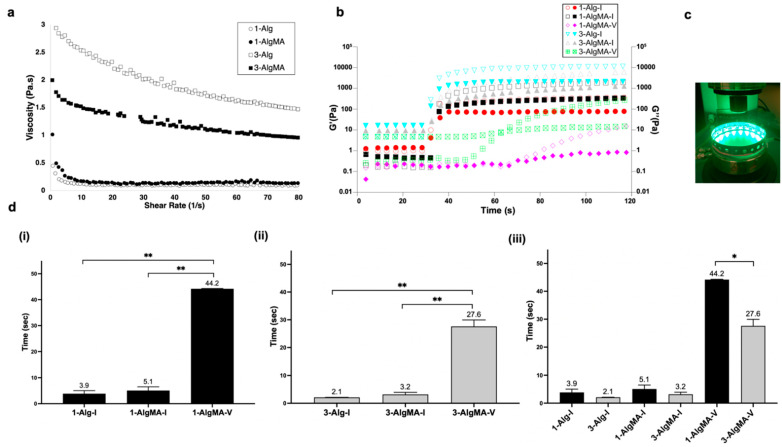
(**a**) Viscosity values versus shear rate for 1 and 3% (*w*/*v*) Alg and AlgMA aqueous solutions. (**b**) Representative curves of storage (G′) and loss (G″) moduli for ionically crosslinked and photo-crosslinked 1 and 3% (*w*/*v*) Alg and AlgMA solutions. Hollow markers indicate G′ values, whereas solid markers indicate G″ values. The crossover of G′ and G″ indicates the initiation of crosslinking of the material to form an elastic component of the hydrogel. The crosslinking process (CaCl_2_ or green light) was initiated 30 s after the start of the test. (**c**) The gelation kinetics for photo-crosslinking were performed on a rheometer with a custom green light setup. (**d**) Gelation times for ionically crosslinked Alg and AlgMA materials and photo-crosslinked AlgMA materials; significant differences were calculated within the (**i**) 1% (*w*/*v*) and (**ii**) 3% (*w*/*v*) hydrogel groups, respectively, and between the (**iii**) 1 and 3% (*w*/*v*) hydrogel groups (* *p* < 0.05, ** *p* < 0.005).

Polymer solution viscosity was measured to determine what physical changes may have occurred to the alginate molecules during methacrylation. Alg and AlgMA solutions, at 1 and 3% (*w*/*v*), respectively, were compared to each other. Representative viscosity versus the shear rate curves for polymer solutions is shown in Figure 3a. At 80 s, the 3% Alg solution viscosity values were higher compared to the 1% and 3% AlgMA solutions at each respective polymer concentration. As indicated by the data, the alginate backbone was degraded during the methacrylation reaction, due to the production of methacrylic acid and a subsequent drop in pH during the chemical reaction. Thus, we hypothesized that a decrease in AlgMA molecular weight, compared to Alg, resulted in lower viscosities. While viscosity was not a limiting factor in this study, it is important nonetheless to mention that any decrease in molecular weight due to degradation may result in decreased crosslinking density, which may influence material performance, e.g., its mechanical properties [33]. 

Gelation kinetics, and the subsequent changes in shear properties during crosslinking, were assessed for each polymer solution (vide supra); the photo-crosslinking was performed on the rheometer with a custom LED device (Figure 3c). Indeed, the differences in viscosity and polymer concentration, and between ionic and covalent crosslinking, resulted in significantly different gelation times for each material group. Gelation times were determined for each material group by the point at which G′ and G″ intersected at the onset of crosslinking. As expected, the gelation times for the 3% solutions were shorter than for the 1% solutions, due to the increased concentration of the polymer and the increased probability of crosslinking occurring between neighboring chains (Figure 3b,d). Upon further comparison, the ionically driven gelation for the AlgMA materials was slower compared to Alg for both polymer concentrations, due to the hypothesized degradation of the AlgMA materials. The resulting increase in gelation time for AlgMA was due to a decreased molecular weight and, thus, a decrease in polymer chain entanglement. In addition, it was noted that the gelation times for the photo-crosslinked materials were significantly longer compared to those for ionic crosslinking. Gelation data were not collected for dual-crosslinked materials (i.e., both ionic and covalent) because the first crosslinking method would determine the gelation point, although we hypothesized that the degree of crosslinking and hydrogel polymer network formation would increase and result in different mechanical properties (vide infra).

### 3.3. Scaffold Structure and Physical Properties

**Figure 4:**
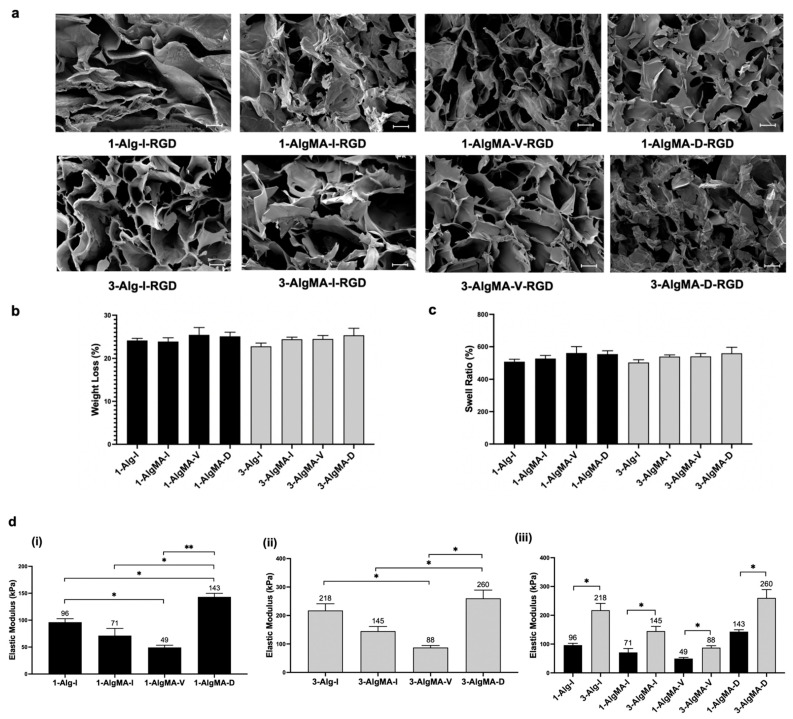
(**a**) SEM images of 1% and 3% (*w*/*v*) Alg and AlgMA hydrogels, formed via ionic, visible light, or dual-crosslinking techniques (scale bar = 40 μm). (**b**) Weight loss and (**c**) swell ratio for alginate-based hydrogels after h in DI water at 37 °C. (**d**) The elastic moduli of the hydrogels were calculated using uniaxial unconfined compression, whereby significant differences were calculated within the (**i**) 1% (*w*/*v*) and (**ii**) 3% (*w*/*v*) hydrogel groups, and between the (**iii**) 1% and 3% (*w*/*v*) samples (* *p* < 0.05, ** *p* < 0.005).

The SEM images, shown in Figure 4a, indicate that the hydrogel pore structure was interconnected, and similar pore size ranges were represented in each group (40–100 µm). While no differences in structure correlated with the material groups overall, there were a few exceptions. The 3-AlgMA-D group exhibited a polymer network with greater density compared to the other materials, while the 1-Alg-I group showed the characteristics of a collapsed network, which may have occurred during dehydration due to its lower structural strength. Slight differences are noted in the different crosslinking techniques used to form the hydrogels. Furthermore, alginate hydrogel scaffolds can be created with pore sizes in the range of 5–200 nm within the walls of the construct, which promotes cell viability by allowing essential molecules such as oxygen, carbon dioxide, urea, glucose, and insulin to travel in and out of the scaffold [33,34]. When comparing the cross-section images of our hydrogels (Figure 4a) to a recent cultured meat study involving ionically crosslinked alginate hydrogels, the images appear similar [35]. While we did not quantify the porosity percentage of our hydrogel, qualitatively speaking, our scaffold showed a similar structure for the ionically crosslinked Alg-RGD group as it used a similar synthesis method to the one employed in the previous study [35]. Ongoing work includes directional crosslinking and defined pore dimensions, where variations in viscosity values can play a significant role in the internal structure of the hydrogel after freezing [36,37]. 

We also conducted an equilibrium water content test and in vitro degradation experiment in DI water at body temperature (37 °C). We assessed whether the polymer concentration and mode of crosslinking played a significant role in the hydrogel weight loss (Figure 4b) or swelling behavior (Figure 4c); there were no significant differences in either weight loss or swell ratio between the different hydrogel groups. Considering the structure and physical properties of the alginate scaffolds were not noticeably nor were they significantly different from one another, the mechanical properties were investigated to see if, indeed, the polymer concentration and crosslinking method had significant effects on the material’s mechanical response. 

### 3.4. Scaffold Mechanical Properties

The compressive elastic moduli were calculated for each material group to determine whether significant differences in the mechanical properties were achieved by adjusting the polymer solution and the crosslinking technique and comparing those values to muscle tissue. A uniaxial compression test was performed due to the nature of the hydrogels. The linear portion of the stress-strain graph, at between 5% and 15% strain, was used to calculate the hydrogel moduli. Comparing the moduli values for the Alg hydrogels to the AlgMA-based hydrogels indicated a decrease in stiffness for both the AlgMA-I and AlgMA-V groups; however, incorporating dual-crosslinking increased the moduli of the AlgMA hydrogels (Figure 4d). Indeed, as shown in the data, covalent crosslinking created a tighter and stiffer network, as represented in the AlgMA-V and AlgMA-D groups. These results occur between groups at both concentrations. The viscosity data for Alg and Alg-MA hydrogels correlates with the compressive moduli of both the 1% and 3% hydrogels. The 1-AlgMA-V was closest to the modulus of native muscle tissue (16–60 kPa); however, the material was difficult to work with, considering the instability of the network [20,21,22]. Visible light crosslinked hydrogels were the best in terms of structure. Ionic crosslinking is the standard method for producing alginate hydrogels, and the use of visible light crosslinking and the presence of photoinitiators have not been investigated as cultured meat scaffolds. In addition, dual-crosslinking is unique in that it incorporates both photo-crosslinking and ionic crosslinking. The net effect of the materials’ stiffness on cell adhesion (vide infra) was qualitatively characterized by selecting the groups with the highest and lowest moduli for both the 1% and 3% alginate hydrogels (Figure 4d). The addition of dual crosslinking confirmed that we can significantly control the mechanical properties by optimizing the polymer solution and the method of crosslinking, which is supported by evidence from the literature [16]. Moving forward with unique and reliable materials for cell toxicity and adhesion experiments, 1% and 3% visible and dual-crosslinked alginate hydrogels were selected for scaffold production.

### 3.5. In Vitro C2C12 Cell Viability

**Figure 5:**
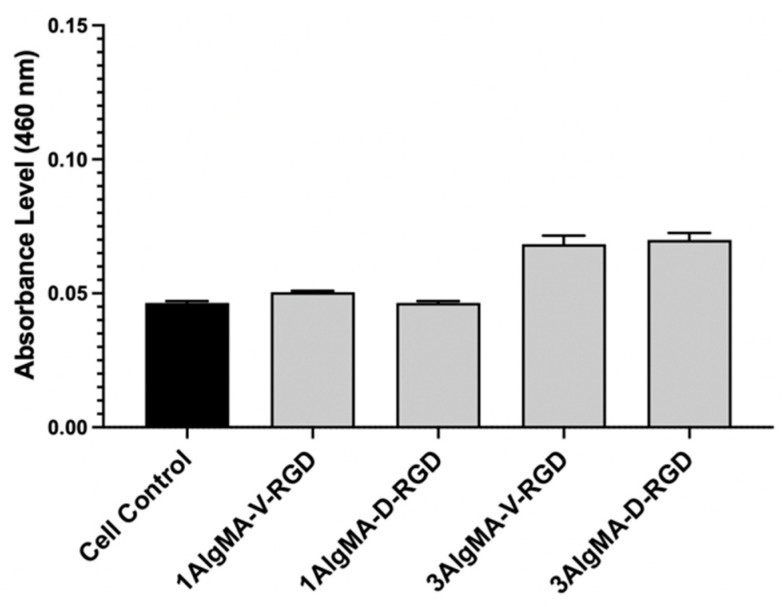
Using a quantitative WST-8 mitochondrial activity assay after 24 h of culture, the scaffold materials were non-toxic to C2C12 cells in vitro.

The murine muscle satellite cells responded favorably to the RGD-modified AlgMA scaffolds, in addition to the scaffolds with the lower moduli. All the material groups indicated higher mitochondrial activity compared to the tissue culture plate control, indicating that all the material groups were not cytotoxic (Figure 5). 

**Figure 6:**
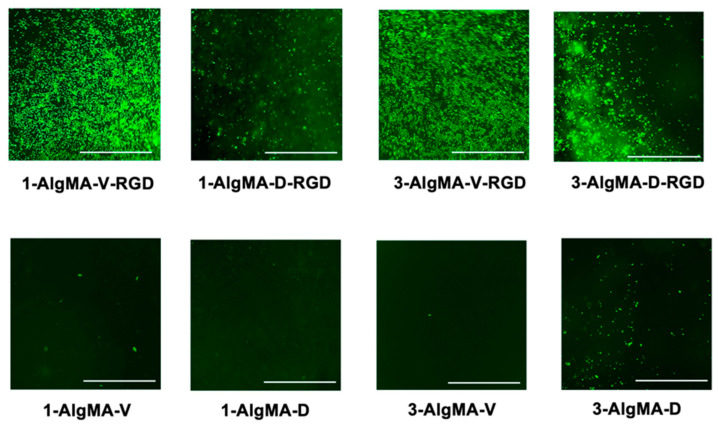
Qualitative fluorescent images of Calcein-AM stained C2C12 cells (bright green) indicated that the scaffolding materials supported cell adhesion and maintained cell viability after 24 h of culture in vitro. The visible-light crosslinked samples indicated higher cell densities compared to the dual-crosslinked samples within the 1 and 3% (*w*/*v*) AlgMA-v-RGD groups (scale bar = 1 mm).

Fluorescent images of viable C2C12 cells were captured to qualitatively characterize the effect of material stiffness on cell adhesion density (Figure 6). Cells remained viable on the RGD-modified materials and were non-adherent on the AlgMA scaffolds without RGD. The 1% and 3% (*w*/*v*) visible light-crosslinked hydrogel scaffolds displayed higher live cell densities compared to the dual-crosslinked samples. These results were expected as the visible light-crosslinked samples exhibited more compliant elastic stiffness values in the range of 49–88 kPa, whereas the dual-crosslinked scaffolds had stiffness values in the range of 143–260 kPa (Figure 4). As described in the literature, the elastic modulus of muscle tissue is in the range of 16–60 kPa [20,21,22]. When seeded onto materials, cells favorably respond to environments and substrates that more closely mimic the properties of the extracellular matrix that makes up the tissue structure [4,5]. Thus, the myoblasts prefer, at least in the short term of 24 h, to adhere and proliferate on softer scaffolds compared to stiffer ones, i.e., those scaffolds exhibiting mechanical properties closer to native tissue. It also appears that a population of cells, which are out of focus in Figure 6, may have migrated within the scaffold, leading to cells at various depths and imaging planes; however, this finding will have to be further investigated in the future, as 3D analysis was outside the scope of this study. For the hydrogel groups without RGD (Figure 6), some cells remained on the surface throughout the 24-hour period of culture. In future studies, we will look at long-term cell viability, proliferation, differentiation, and migration. Herein, we showed that our material composition was not cytotoxic to C2C12 cells, which correlates with the published data indicating that AlgMA and AlgMA-RGD are innocuous [14]. 

## 4. Conclusions

In this study, we quantitatively and qualitatively show the effect of visible light and dual-crosslinked alginate-based hydrogel scaffolds on the viability and adhesion response of muscle satellite cells. By adjusting the polymer concentration and the crosslinking methodology, the scaffold’s mechanical properties were optimized to fall within the physiological range. Thus, the utility of our materials is beneficial not only for the tunability of the material properties but also for the future capability of producing more complicated scaffolds with various sections of differing properties. Remarkably, the porous structure and physical properties of the hydrogels were not appreciably altered, while the compressive moduli were significantly altered using different crosslinking approaches. This proof-of-concept study verified the use of mechanical properties optimization for the further development of cultured meat scaffolds. Future work will focus on cell differentiation within alginate-based materials of varying stiffness. 

## Figures and Tables

**Table 1:** Hydrogel scaffold groups comprising alginate in its non-modified (Alg) and chemically modified (AlgMA) forms. The material groups selected for this study differed with regard to polymer concentration, the method of crosslinking, and cell adhesion ligand conjugation (i.e., RGD). Material group labels are identified in the far-right columns.

Polymer % (*w*/*v*)	Material	Crosslinking Type	Hydrogel Groups	Hydrogel Groups + Ligand
1	Alginate	Ionic	1-Alg-I	1-Alg-I-RGD
		Ionic	1-AlgMA-I	1-AlgMA-I-RGD
	Methacrylated alginate	Visible	1-AlgMA-V	1-AlgMA-V-RGD
		Dual	1-AlgMA-D	1-AlgMA-D-RGD
3	Alginate	Ionic	3-Alg-I	3-Alg-I-RGD
		Ionic	3-AlgMA-I	3-AlgMA-I-RGD
	Methacrylated alginate	Visible	3-AlgMA-V	3-AlgMA-V-RGD
		Dual	3-AlgMA-D	3-AlgMA-D-RGD

## Data Availability

The data presented in this study are available on request from the corresponding author.
